# Design of a Template-Based Electrophoretically Assisted Micro-Ultrasonic Machining Micro-Channel Machine Tool and Its Machining Experiment

**DOI:** 10.3390/mi14071360

**Published:** 2023-06-30

**Authors:** Haishan Lian, Linpeng Zhang, Xiaojun Chen, Cuiyuan Deng, Yuandong Mo

**Affiliations:** 1 School of Mechanical and Electrical Engineering, Lingnan Normal University, Zhanjiang 524048, China; 2 School of Mechanical and Electrical Engineering, Guangdong University of Technology, Guangzhou 510006, China

**Keywords:** micro-channel machining, electrophoretically assisted machining, micro-ultrasonic machining, template, machine tool design, machining experiment

## Abstract

In order to achieve the high-precision and high-efficiency machining of micro-channels for hard and brittle materials, the authors innovatively proposed a new technology called template-based electrophoretically assisted micro-ultrasonic machining (TBEPAMUSM). This technology transfers the micro-channel shape punch-pin to the workpiece material through micro-ultrasonic machining to form a micro-channel. At the same time, it uses the electrophoretic properties of ultra-fine abrasive particles to ensure the existence of abrasive particles in the machining area by applying a DC electric field. According to the new technology machining principle, a machine tool of TBEPAMUSM was designed and developed. The machine tool hardware adopts a C-shaped structure, including a marble platform, an ultrasonic vibration system, a micro three-dimensional motion platform, a working fluid tank, and a pressure sensor. The machine tool intelligent control system is developed based on LabVIEW, including the initialization module, fast positioning module, constant force tool setting module, constant force control machining module, and real-time coordinate display module. Micro-channels with different structures are machined on single-crystal silicon and soda-lime glass using the designed machine tool and the developed control system. The results show that: when electrophoresis assistance is applied in machining, the edge chipping phenomenon of the micro-channel is significantly reduced, the surface roughness is reduced by about 20%, and the machining efficiency is increased by about 4%.

## 1. Introduction

With the development of microfabrication technology, microfluidic chips and micro-structures are widely used in the core components of industrial equipment such as machinery, aerospace, and biochemical medicine. Their performance determines the quality of the entire equipment [1,2,3,4,5,6,7,8,9,10]. The micro-channel is the core structure of the microfluidic chip, and its machining accuracy and molding quality determine the performance of the microfluidic chip. Hard and brittle materials have high-temperature resistance, small thermal expansion coefficients, high strength, wear resistance, good chemical stability, and good heat dissipation performances, so they are one of the ideal materials for preparing microfluidic chips [11,12,13,14]. However, high-efficiency and high-precision machining methods for hard and brittle materials have always been a research difficulty in machining and manufacturing [15]. At present, micro-channels are often prepared by chemical etching [16], laser machining [17], micro-milling [18,19], casting molding [20], and other machining technologies.

Chen Xiaoxiao [21] used laser machining to machine a micro-texture with specific characteristics on the workpiece’s surface to discretize the workpiece’s surface and then performed rotary ultrasonic machining to machine a micro-groove with a width of 177 μm and a depth of 83 μm. The surface roughness of the micro-grooves was 1.33 μm. Basem [22] used rotary ultrasonic machining to machine micro-channels in biolox forte ceramic. The micro-channels with a good surface finish were obtained, with a cross-section width of 800 μm, a depth of 300 μm, an Ra of 0.21 μm, and an Rt of 2.3 μm.

Compared with other micro-structure machining methods, micro-ultrasonic machining does not depend on the material’s conductivity, the macro force of the tool on the workpiece is small, and the thermal influence is small. So, it is often used to manufacture thin slices and hard and brittle materials. However, micro-ultrasonic machining also has limitations, and scholars from various countries have made different explorations and research.

Zhao [23] found that slight lateral vibrations at the end of the tool are unavoidable, leading to over-cutting and edge-chipping. He proposed to ensure high surface integrity by applying a protective coating on the substrate. The results show that this measure can improve the quality of micro-ultrasonic machining, and the surface integrity index is improved by 65.85% under the optimal experimental parameters.

Tateishi [24,25,26] studied the cause of micro holes edge-chipping in micro-ultrasonic machining and attributed it to the result of the tool head directly hammering the workpiece. The abrasive particles in the narrow machining area will be vibrated out of the machining area by the high-frequency vibrating tool so that there will be no abrasive particles in the machining area, and the phenomenon was observed by CCD [27]. 

Electrophoretically assisted micro-ultrasonic machining is to give full play to the electrophoretic properties of ultra-fine abrasive particles. By applying an electric field, the ultra-fine abrasive particles swim toward the machining area to ensure the existence of abrasive particles in the machining area and improve the surface quality and machining efficiency [28]. In this paper, based on the electrophoretic characteristics of ultrafine abrasive grains and the advantages of ultrasonic machining hard and brittle materials, a template-based electrophoretically assisted micro-ultrasonic machining machine tool of a micro-channel and its intelligent control system was developed, and machining experiments were carried out. The difference between this paper and already published work [28] is that the tool used in this paper is a template tool, which can be machined into a micro-channel at one time for the rapid, mass production of the same-shaped microchannels. The tool used in the published article is a cylindrical micro-tool, and the research is on the influence of various machining parameters on the material removal rate and the quality of micro-hole machining.

## 2. Principle of Template-Based Electrophoretically Assisted Micro-Ultrasonic Machining

**Figure 1:**
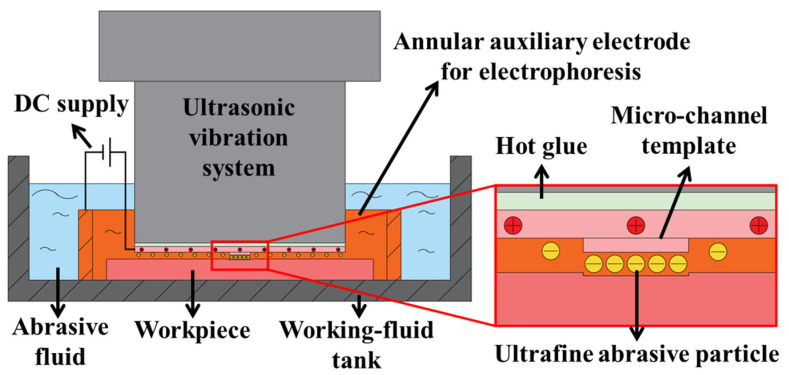
Schematic diagram of template-based electrophoretically assisted micro-ultrasonic machining.

Template-based electrophoretically assisted micro-ultrasonic machining is to transfer the micro-channel shape punch to the workpiece through micro-ultrasonic machining at one time. At the same time, using the electrophoretic characteristics of ultra-fine abrasive grains, the presence of abrasive particles in the machining area is ensured by applying a DC electric field. The machining principle is shown in Figure 1. The ultrasonic vibration system uses the ultrasonic transducer to convert the high-frequency electrical signal generated by the ultrasonic power supply into ultrasonic vibration. After the ultrasonic horn amplifies the vibration, the template tool installed on the transducer is prompted to vibrate at the corresponding frequency. Furthermore, they drive the abrasive particles in the working fluid to vibrate at a high frequency to achieve impact and polishing on the workpiece. The template tool is connected to the positive pole of the DC power supply, and the annular auxiliary electrode is connected to the negative pole of the DC power supply. At this time, the center of the annular auxiliary electrode will form an auxiliary electric field. The electrophoretic characteristics of ultrafine abrasive particles are used in machining. The ultrafine abrasive particles swim to the machining area under the action of the auxiliary electric field to ensure the existence of abrasive particles in the machining area, thereby improving the machining quality and machining efficiency of micro-channels.

## 3. Design of an Experimental Machine Tool

**Figure 2:**
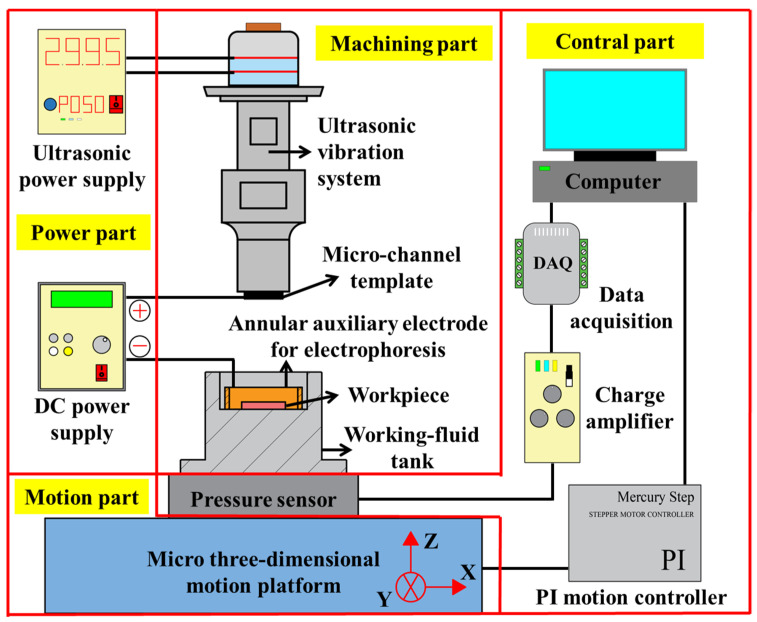
Schematic illustration of the experimental setup.

According to the principle of template-based electrophoretically assisted micro-ultrasonic machining, the experimental device shown in Figure 2 is designed, which consists of four parts: the power part, machining part, control part, and motion part. The power part includes an ultrasonic power supply and a DC power supply. The ultrasonic power supply supplies energy to the ultrasonic vibration system. The positive pole of the DC power supply is connected to the template tool, and the negative pole is connected to the annular auxiliary electrode. The machining part comprises an ultrasonic vibration system, a template tool, a workpiece, an annular auxiliary electrode, and a working fluid tank. The ultrasonic vibration system transmits the ultrasonic amplitude to the template tool through a piezoelectric ceramic sheet and ultrasonic horn. The template tool is a pre-processed micro-channel shape punch. The workpiece is a hard and brittle sheet material. The annular auxiliary electrode is a copper ring. After the DC power is turned on, an auxiliary electric field will be formed between the annular auxiliary electrode and the tool. The working fluid tank is filled with a solution mixed with ultrafine abrasive particles. It has a leveling function, which can minimize the parallelism error generated when installing the workpiece. The control part includes an industrial computer, data acquisition card, charge amplifier, pressure sensor, and PI motion controller. The real-time feedback voltage signal of the pressure sensor passes through the charge amplifier and is then sent back to the industrial computer by the data acquisition card. The industrial computer accurately controls the feed movement of the micro three-dimensional motion platform through the PI motion controller according to the real-time feedback pressure. The motion part is a micro three-dimensional motion platform, and the micro three-dimensional motion platform performs feed motion during machining.

### 3.1. Key Components Design of the Machine Tool

The critical components of this machine tool include a vertical sliding table, micro three-dimensional motion platform, pressure sensor, ultrasonic vibration system, working fluid tank, and template tool. The hardware of the motion system adopts the large-displacement moving parts of the vertical sliding table, which is combined with the small-displacement motion system of the micro three-dimensional motion platform.

#### 3.1.1. Design of a Vertical Sliding Table

**Figure 3:**
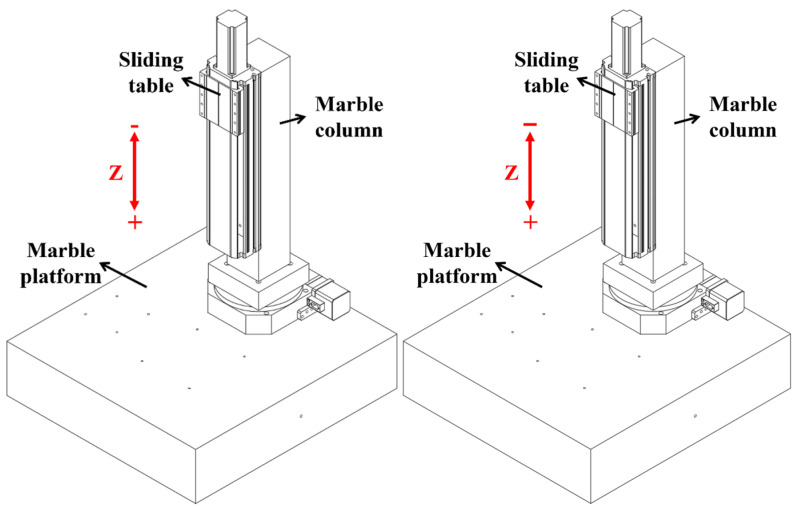
Vertical sliding table.

The three-dimensional diagram of the vertical sliding table is shown in Figure 3, composed of a marble column + sliding table. The marble column is installed on the marble platform. The sliding table adopts the structure of a screw + double guide rail. The selected servo motor has a self-locking function.

#### 3.1.2. Design of a Micro 3D Motion Platform

**Figure 4:**
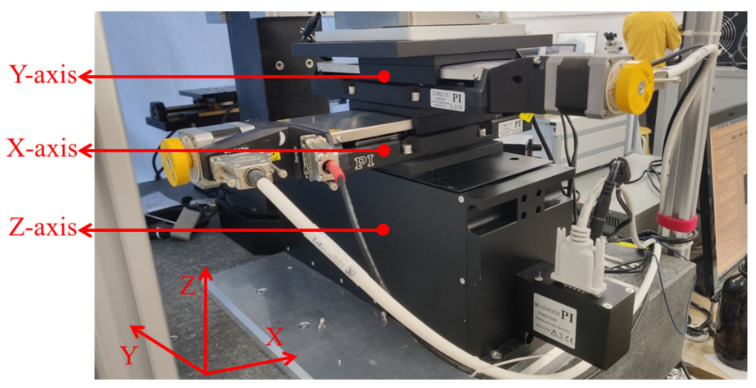
Micro three-dimensional motion platform.

The micro three-dimensional motion platform is shown in Figure 4. It is installed and fixed on the marble platform. The strokes of the X-axis, Y-axis, and Z-axis are 102 mm, 102 mm, and 25 mm, and the minimum resolution of each axis is 0.1 μm.

#### 3.1.3. Pressure Sensor

**Figure 5:**
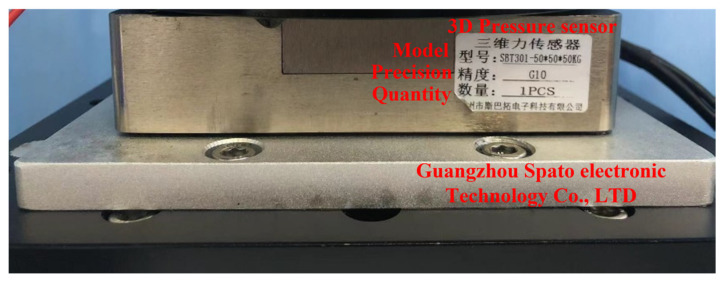
Pressure sensor.

The micro three-dimensional motion platform is shown in Figure 5. The three-axis measuring range is 500 N, and the comprehensive error is ≤±1%. It is used for constant force tool setting before machining and real-time force feedback during machining.

#### 3.1.4. Design of an Ultrasonic Vibration System

**Figure 6:**
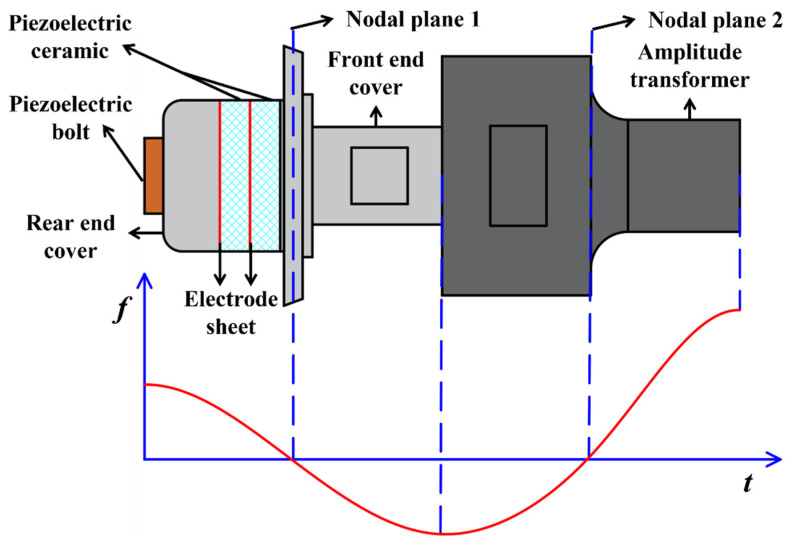
Structure of an ultrasonic vibration system.

The energy of the ultrasonic transducer system is generated by piezoelectric ceramics and transmitted between the front cover and the ultrasonic horn, the horn, and the tool. The transducer element in the piezoelectric transducer adopts an axially polarized piezoelectric ceramic ring. The piezoelectric bolts are used to fix the rear end cover, electrode piece, and front end cover, as shown in Figure 6. The length of the entire vibration system is one wavelength. The ultrasonic transducer and the horn are half a wavelength each. The first nodal plane is designed in the flange of the ultrasonic transducer for fixing on the ultrasonic vibration system mounting base. The second nodal plane is designed at the horn, that is, the whole vibration system is divided into four quarter wavelengths by two nodal planes; the first quarter wavelength is composed of the rear end cover and piezoelectric ceramic sheet, the second quarter wavelength is the front cover, and the third quarter wavelength and the fourth quarter wavelength are the front and rear parts of the horn, respectively. The material of the rear end cover is 45# steel, the front end cover is duralumin, and the material of the horn is titanium alloy.

#### 3.1.5. Design of a Working Fluid Tank

**Figure 7:**
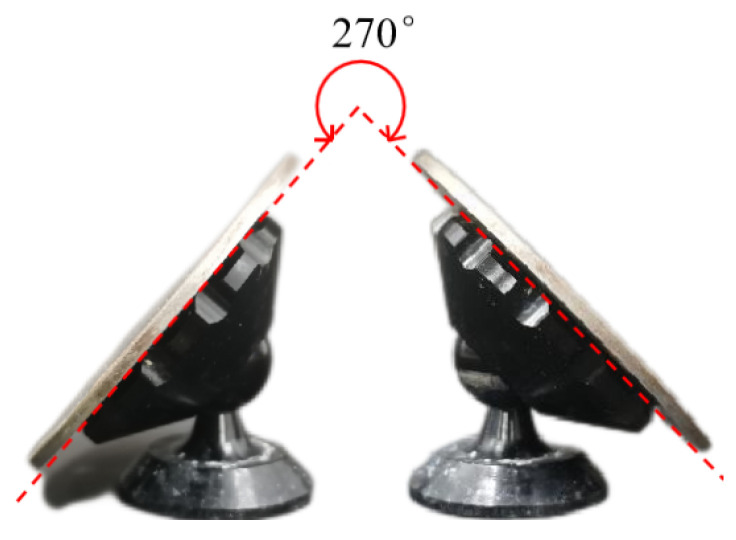
Leveling stents.

To ensure the micro-channels’ structural integrity and average depth, it is necessary to ensure the parallelism between the template tool and the workpiece. Given the above problems, the author added a leveling device in the working fluid tank. The device can be rotated 360° in the horizontal direction and adjustable in the vertical direction from 0 to 270°. The actual picture is shown in Figure 7.

**Figure 8:**
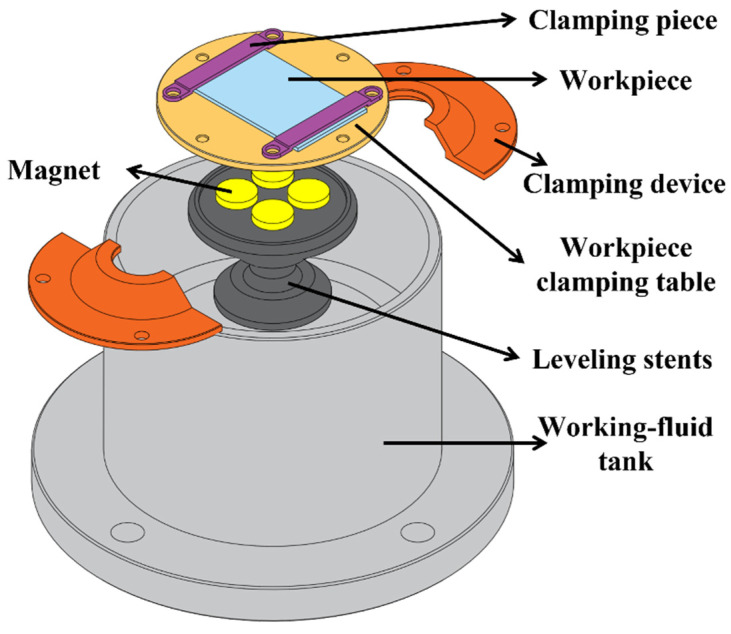
Working fluid tank exploded view.

**Figure 9:**
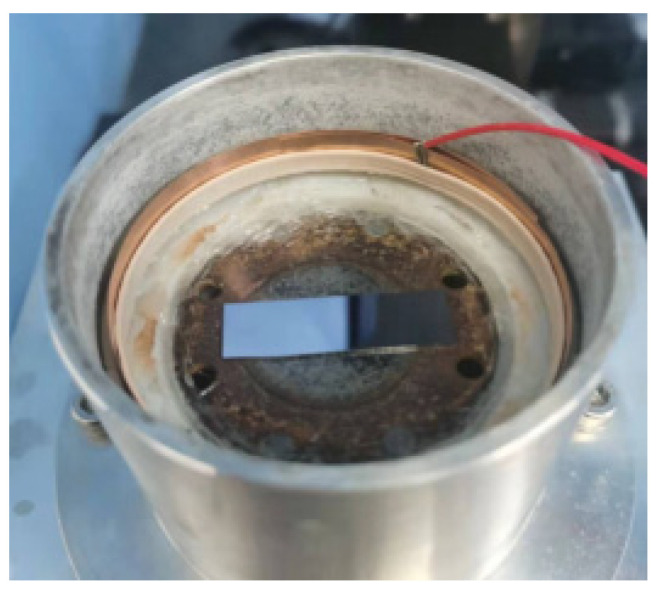
Working fluid tank.

The leveling stents are fixed and clamped on the bottom of the working fluid tank with two clamping devices. The workpiece clamping table is attracted to the top of the leveling stents through four powerful magnets on the bottom annular boss, and the clamping piece and bolts fix the two ends of the workpiece. The exploded views are shown in Figure 8, and the physical figure is shown in Figure 9.

#### 3.1.6. Design of a Template Tool

The template tool material used in this experiment is rugged aluminum with good mechanical properties, high anti-fatigue strength, high electrical conductivity, and easy processing. The relevant parameters are shown in Table 1.

**Figure 10:**
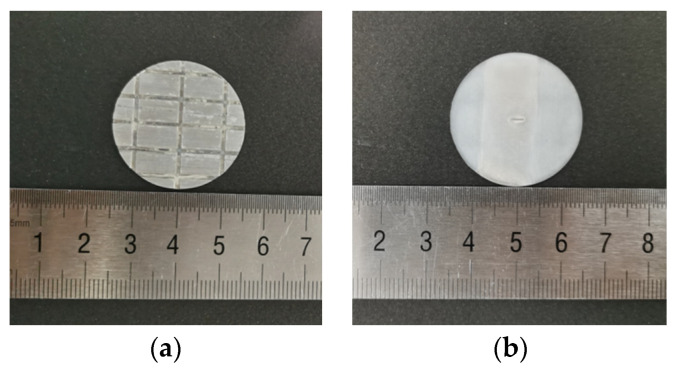
Micro-channel template: (**a**) back of the tool; (**b**) front of the tool.

Its structure is round and thin, and the diameter is equal to the diameter of the ultrasonic horn, as shown in Figure 10. The purpose of machining the whole row of grooves on the back of the template tool is to better bond with the ultrasonic vibration system.

**Figure 11:**
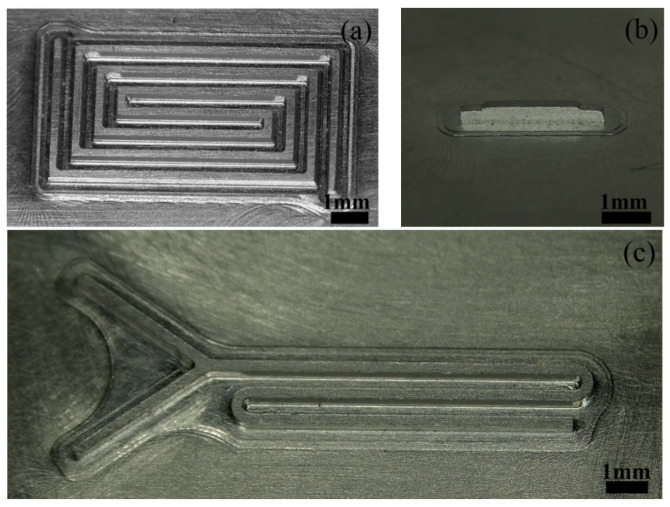
Physical drawing of the tool protrusion structure: (**a**) back-shaped array; (**b**) step-shaped; (**c**) Y-curved runner.

According to the principle of template-based electrophoretically assisted micro-ultrasonic machining, and to meet the needs of micro-channels used in different situations, three different micro-channel shapes are designed in this paper. They are a back-shaped array micro-channel (Figure 11a), a step-shaped straight groove micro-channel (Figure 11b), and a Y-curved runner micro-channel (Figure 11c).

**Figure 12:**
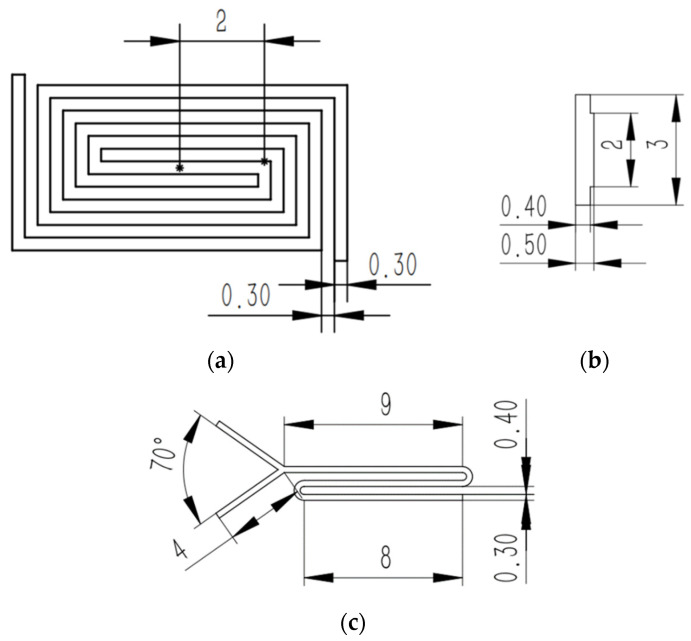
Protruding structure and dimension design of the tool: (**a**) back-shaped array; (**b**) step-shaped; (**c**) Y-curved runner.

The size of the back-shaped array micro-channel is shown in Figure 12a, and the height of the micro-bulge of the template tool is 0.5 mm. The size of the step-shaped straight groove micro-channel is shown in Figure 12b, and the width of the micro-bulge of the template tool is 0.3 mm. The size of the Y-curved runner micro-channel is shown in Figure 12c, and the micro-bulge height of the template tool is 0.5 mm.

#### 3.1.7. Machine Tool of TBEPAMUSM

**Figure 13:**
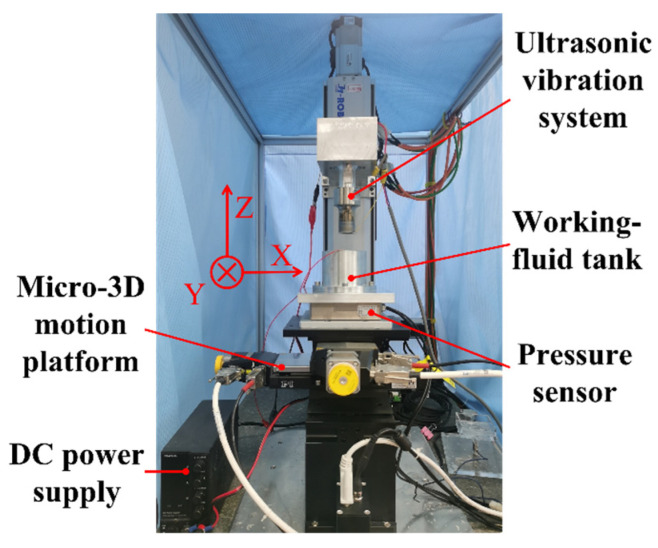
Physical map of the template electrophoretically assisted micro-ultrasonic machining device.

The electrophoretically assisted micro-ultrasonic machining machine tool of the micro-channel is shown in Figure 13. The pressure sensor is installed on the micro three-dimensional motion platform through the adapter plate, the working fluid tank is installed on the pressure sensor, the template tool is affixed to the lower end face of the ultrasonic vibration system with 3M double-sided adhesive, and the ultrasonic vibration system is installed on the vertical sliding table through the supporting arm.

### 3.2. Control System Design of the TBEPAMUSM Machine Tool

**Figure 14:**
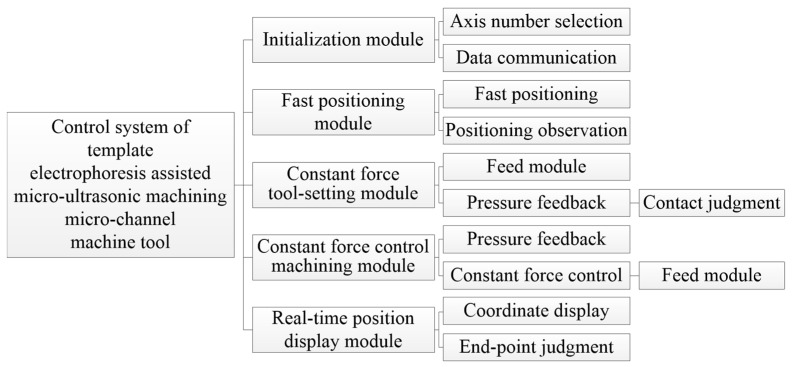
Template electrophoresis-assisted micro-ultrasonic machining micro-channel machine control system architecture.

The control system architecture of the electrophoretically assisted micro-ultrasonic machining micro-channel machine tool is shown in Figure 14. It includes an initialization module, fast positioning module, constant force tool setting module, constant force control machining module, and real-time position display module.

The initialization module powers up and resets each shaft during the specific operation. Then, the tool setting module is switched to, and the program origin is determined through fast positioning and constant force tool setting. Finally, the constant force control machining module is switched to to carry out the TBEPAMUSM of the workpiece. The real-time position display module feeds back the current machining coordinates of each axis in real time.

The function of the initialization module is to establish the communication between the LabVIEW and the micro three-dimensional motion platform and automatically move to the origin of the machine tool after establishing the communication.

The function of the fast-positioning module is to control the axis of the micro three-dimensional motion platform to move to the target position at a faster speed. When the tool is still a long distance from the workpiece, the artificial control of the workpiece fast approaching the tool is the safest, quickest, and most convenient.

The real-time position display module is independent of other modules. It is located in the outermost layer of the whole control system architecture, which ensures that the current axis coordinates can be displayed in real time regardless of switching to any module.

#### 3.2.1. Constant Force Tool Setting Module

**Figure 15:**
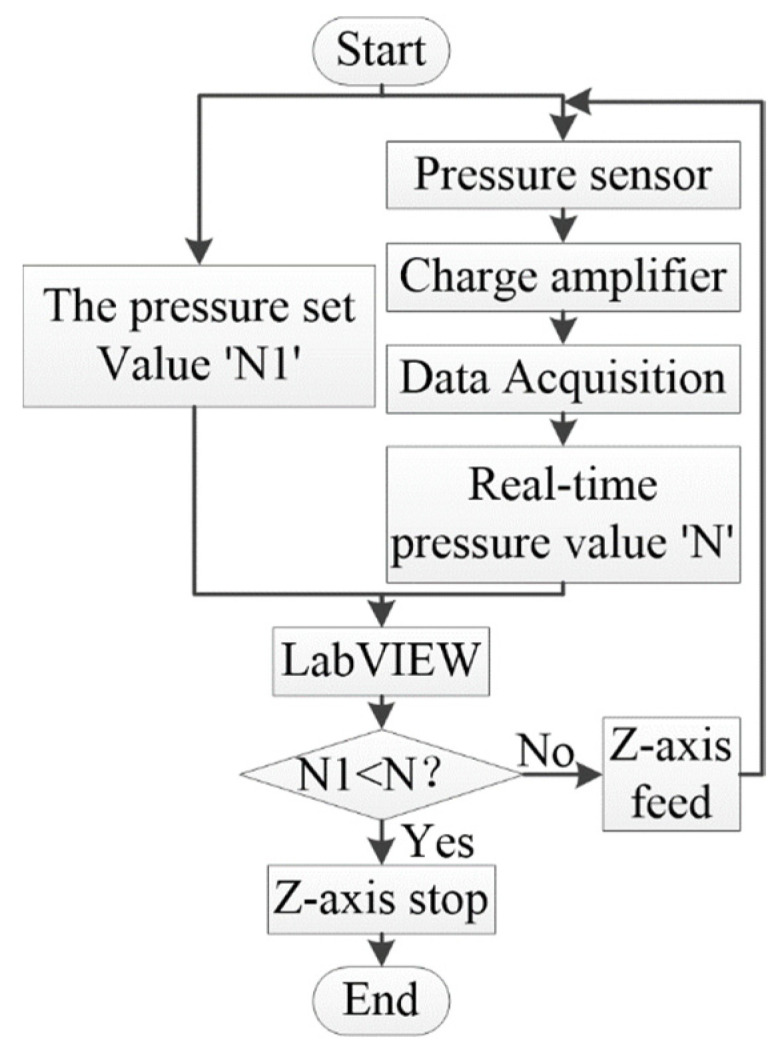
Flow chart of the constant force tool-setting module.

The flow chart of the constant force tool setting is shown in Figure 15. The pressure sensor provides real-time feedback on the contact force between the tool and the workpiece, and the control system compares the real-time pressure value with the pressure set value. When the real-time pressure value is greater than or equal to the pressure setting value, the LabVIEW program immediately brakes the Z axis of the micro three-dimensional motion platform, and the current point can be regarded as the program origin. The pressure set value is usually set 0.1 N higher than the real-time pressure value.

#### 3.2.2. Constant Force Control Machining Module

**Figure 16:**
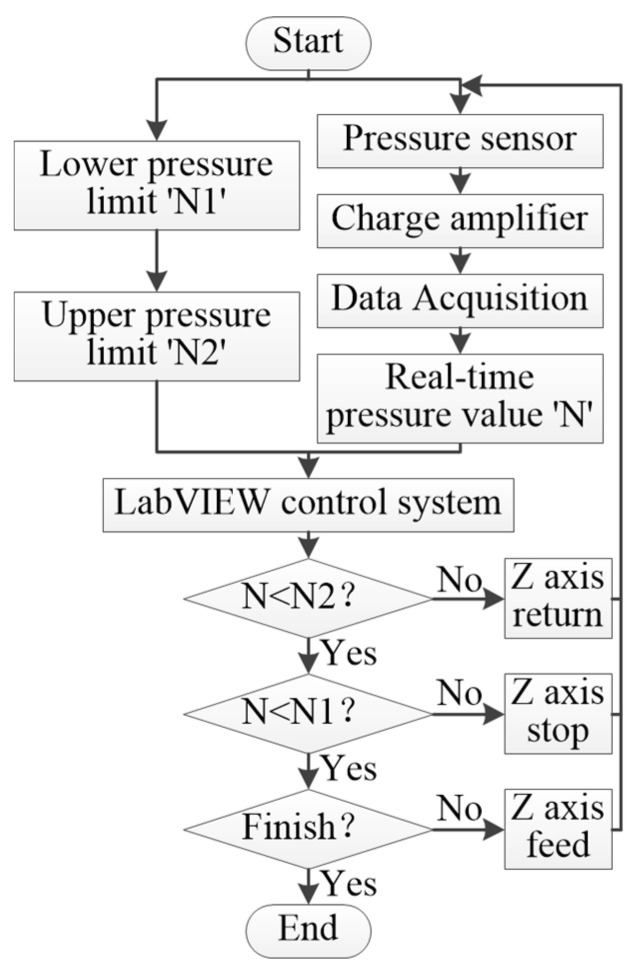
Flow chart of the constant force control machining module.

The constant force control machining module is carried out after the constant force tool setting module because the template-based electrophoretically assisted micro-ultrasonic machining requires a slight force contact between the tool and the workpiece. Therefore, the purpose of this module is to control the pressure between the tool and the workpiece in a constant range. The Z axis will retreat if the pressure exceeds the upper-pressure limit value. It will feed if it is less than the lower pressure limit value. Furthermore, it will remain still if it is in the pressure range. The flow chart of constant force control machining is shown in Figure 16.

#### 3.2.3. The Interface of the Control System

**Figure 17:**
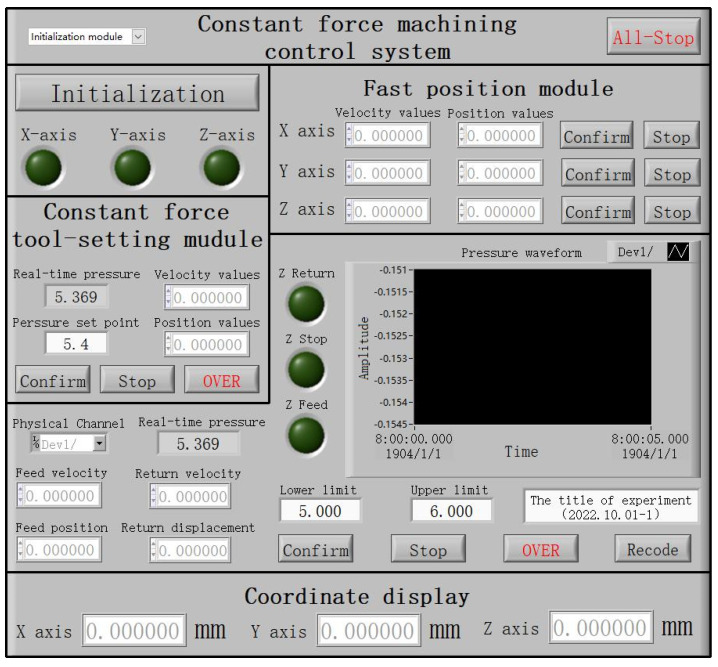
The interface of the template electrophoretically assisted micro-ultrasonic machining control system.

The program control interface of template-based electrophoretically assisted micro-ultrasonic machining is shown in Figure 17. After running the initialization module, the user can select different motion modules by selecting the drop-down menu of the motion control selection module. When one of the motion modules is selected, the control buttons of the other motion modules will not work.

## 4. Machining Experiment and Result Analysis

The silicon wafer used in the experiment is n-type silicon with a <100> orientation provided by Zhejiang Lijing Silicon Material Co., Ltd. (Quzhou City, China), the size is 38 mm × 12 mm × 0.7 mm, and the resistivity is 2 Ω cm~5 Ω cm. Soda-lime glass has a size of 38 mm × 12 mm × 1 mm, provided by Changshu City Xinzhuang town Yi Jian Glass shop (Changshu City, China).

The Morse hardness of the diamond particles used in the experiment is 10.0, and the particle size is 10,000 mesh, provided by Zhecheng County Hua Drill Superhard Material Co., Ltd. (Shangqiu City, China). The gray-green silicon carbide abrasive particles used in the experiment have a Morse hardness of 9.5, wear, and high-temperature resistance, provided by Qinghe County Kete New Material Technology Co., Ltd. (Xingtai City, China). Diamond particles are mixed with silicon carbide abrasive particles with a mixing ratio of 1:19.

### 4.1. Experimental Study on Micro-Channel Machining

A laser scanning confocal microscope is the experimental equipment used in this paper, which can measure the surface roughness, length, width, and depth of the microchannel. The calculation of the material removal rate is shown in Equation (1). The length is ‘*a*’, the width is ‘*b*’, and the depth is ‘*h*’. During the experiment, each machining was for 6 min, that is, the machining time ‘*T*’ was 6 min.

(1) $$MRR=\frac{a\times b\times h}{T}$$

In the micro-channel machining experiment, the experimental conditions are as follows: ultrasonic power 70%, average particle size 18 μm, abrasive particles concentration 18%, DC voltage 40 V, average machining force 1.5 N, average width of the tool bulge structure 300 μm, feed speed 0.01 mm/s, and retreat speed 0.02 mm/s. Lift the knife every other minute to facilitate the removal of chips and let the abrasive particles into the machining area.

The results show that the coverage of the back-shaped array micro-channel is 8 mm × 4 mm, that of the Y-curved runner micro-channel is about 10.5 mm × 4.6 mm, and that of the step-shaped micro-channel is 3 mm × 0.3 mm.

**Figure 18:**
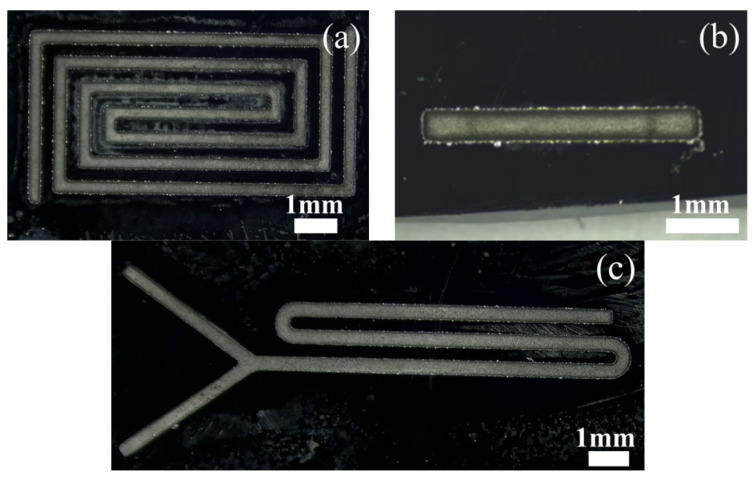
Microscopic magnification of the silicon-based microfluidic channel: (**a**) back-shaped array; (**b**) step-shaped; (**c**) Y-curved runner.

**Figure 19:**
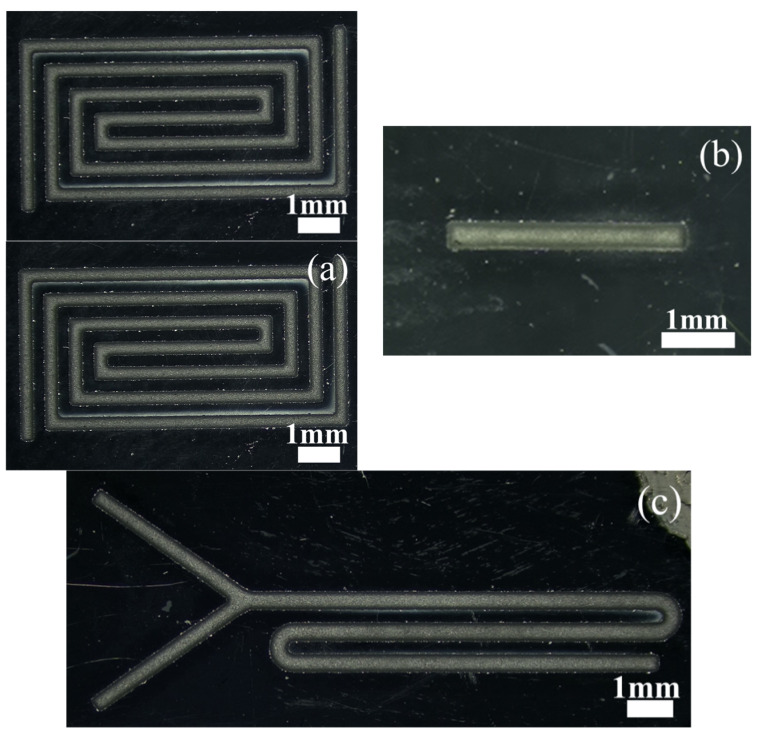
Microscopic magnification of the soda-lime glass-based microfluidic channels: (**a**) back-shaped array; (**b**) step-shaped; (**c**) Y-curved runner.

Figure 18 is a micro-magnification of micro-channels machined on silicon wafers. Figure 19 is a micro-enlarged view of a micro-channel machined on soda-lime glass.

**Figure 20:**
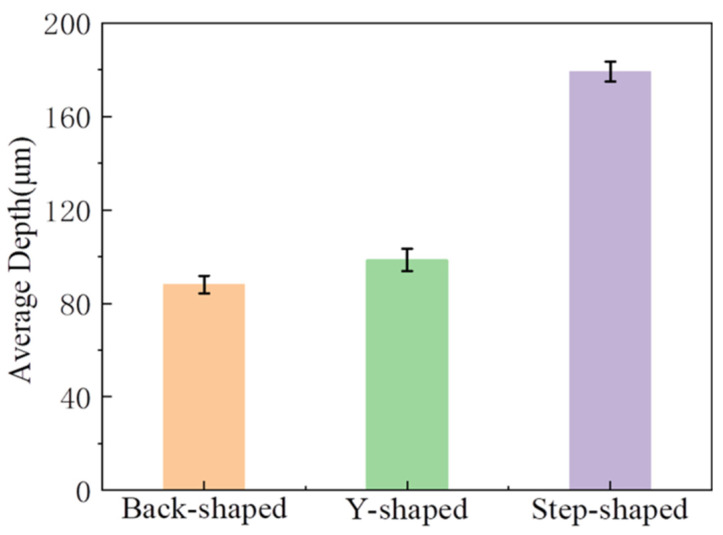
Depth comparison chart of different shapes.

Because the volume of the micro-channel with complex shapes is difficult to calculate, and it is difficult to obtain relatively accurate data, the average depth of the micro-channel is used as the evaluation index of machining efficiency. The comparison of different shapes and depths is shown in Figure 20. It is observed from the diagram that when machining micro-channels of different shapes on non-conductive monocrystalline silicon, the machining rate of stepped micro-straight grooves is the highest. This is because, among the three shapes, the contact area between the step-shaped micro-straight groove micro-bulge structure and the workpiece is the smallest. Under the same energy, the larger the cross-sectional area of the micro-bulge structure, the smaller the tool end amplitude, and the impact of the wear particles on the workpiece decreases, which affects the machining rate. Furthermore, when the cross-sectional area is larger, it is more difficult for the wear particles to enter the machining area evenly. The utilization rate of wear particles in the working fluid decreases, and the machining rate decreases.

**Figure 21:**
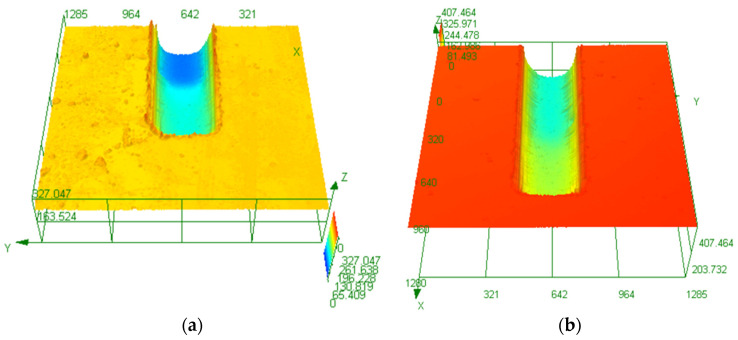
Internal morphology of the stepped microchannel: (**a**) Si; (**b**) glass.

In order to observe the actual shape of the micro-channel, the internal shape of the step-shaped micro-channel is analyzed. The three-dimensional figure is shown in Figure 21. By outlining the steps, it can be found that the step-shaped micro-channels formed on the single-crystal silicon wafer substrate and the soda-lime glass substrate are all rounded transitions. Outlining the steps shows that the step-shaped micro-channels formed on the single-crystal silicon wafer substrate and the soda-lime glass substrate are all rounded transitions. At the same time, the tool protrusion structure is not provided with rounded corners. We found that this is related to the irregularity of the movement of abrasive particles in ultrasonic machining, and the sharp corners of the tool’s convex structure are quickly impacted and worn by abrasive particles.

**Figure 22:**
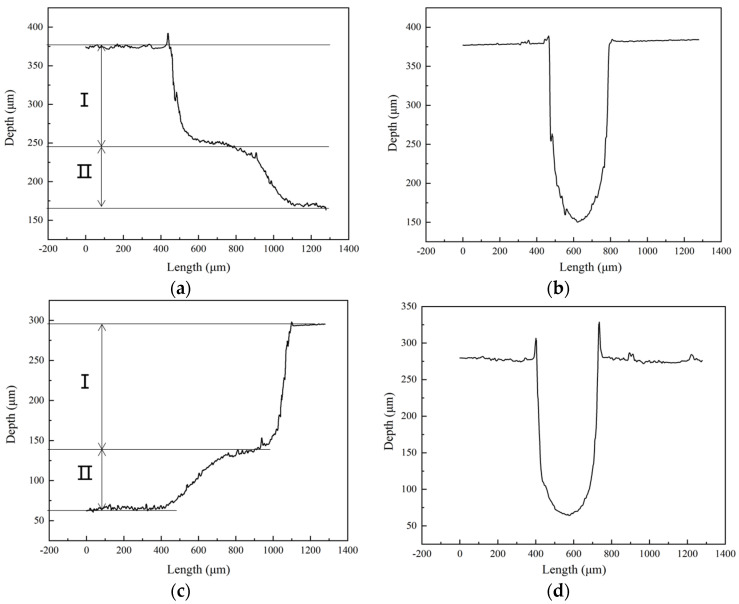
Internal profile curves of stepped-trapezoidal microchannels: (**a**,**b**) based on Si; (**c**,**d**) based on glass.

As shown in Figure 22, the height of the second step of the silicon substrate is about 80 μm, and in the soda-lime glass substrate, it is about 74 μm, but in the template tool, it is 100 μm. We found that this is because, after the step is formed, the abrasive particles in the working fluid easily accumulate at the first step due to the constraints of the raised step structure of the tool and the impact on the junction of the first and second steps under the action of ultrasonic vibration such that the height difference of the second step is minor. “I” is the first step, “II” is the second step.

**Figure 23:**
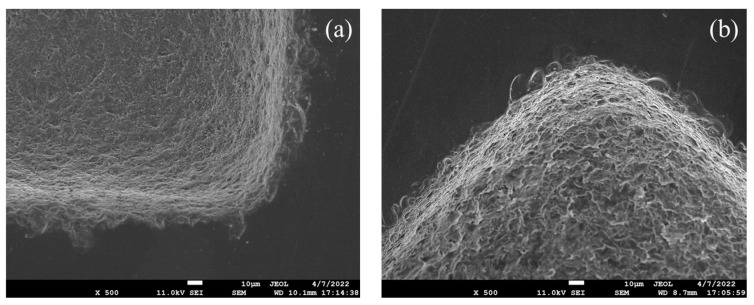
Detailed view of the right angle of the microfluidic channels: (**a**) based on single-crystal silicon wafer; (**b**) based on soda-lime glass.

Microscopic morphology at the right angle of the micro-channel is shown in Figure 23. It can be seen that, whether it is a single-crystal silicon substrate or a soda-lime glass substrate, the ‘rounding effect’ is obvious, which is related to the characteristics of ultrasonic machining and the wear of the convex structure of the tool. By observing its morphology, we found that the edge cracks at the entrance of silicon-based micro-channels are wide, and edge-chipping is easier to connect into pieces. In contrast, the edge-chipping depth at the entrance of glass-based micro-channels is relatively deep and not connected into pieces.

**Figure 24:**
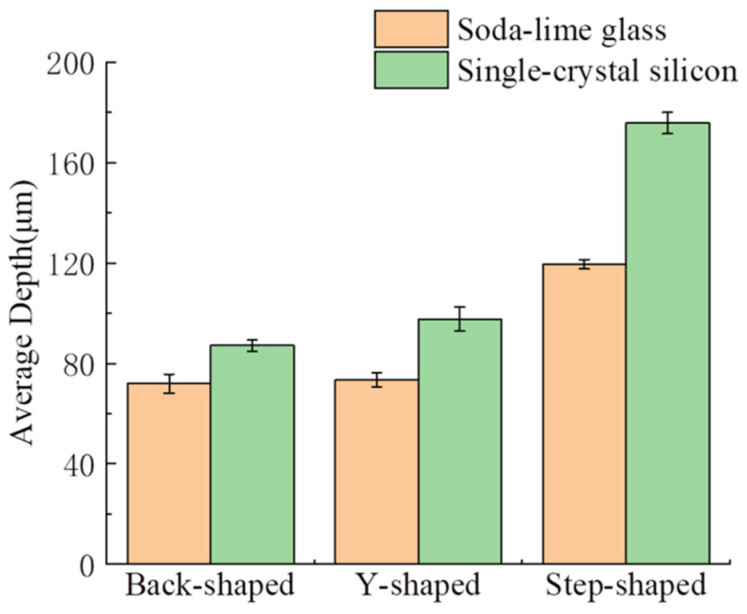
Depth comparison chart of different materials.

Figure 24 compares the depth of micro-channels of different shapes machining on single-crystal silicon and soda-lime glass. It is found that the efficiency of machining micro-channels on single-crystal silicon is better, which shows that the mechanical properties of materials have a more significant impact on the machining efficiency of materials. The fracture toughness of single-crystal silicon is about 0.82 MPa·m^1/2^, and that of soda-lime glass is about 0.8~0.9 MPa·m^1/2^. Under the same machining conditions, the higher the fracture toughness of the material, the smaller the surface layer removal fragmentation and the more complex the material removal.

### 4.2. Comparative Experiment

Table 2 shows the surface roughness and material removal rate of micro-groove machining with or without electrophoretically assisted micro-ultrasonic machining. It can be seen from Table 1 that when an electric field is applied in the working fluid tank, the surface roughness of the micro-groove can be effectively reduced. This is because the ultra-fine abrasive particles in the working fluid swim toward the machining area under the electric field traction. Under ultrasonic vibration, they can polish small pits and protrusions, thereby reducing the surface roughness. Compared with micro-ultrasonic machining, electrophoretically assisted micro-ultrasonic machining positively improves the material removal rate. Therefore, the impact of electrophoretically assisted machining is mainly reflected in the improvement of the surface roughness and edge chipping.

**Figure 25:**
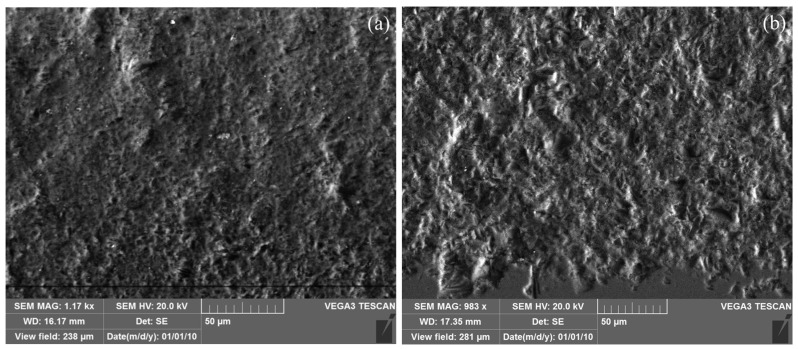
Micro-morphology of the micro-grooves surface under different processing methods: (**a**) electrophoretically assisted micro-ultrasonic machining; (**b**) micro-ultrasonic machining.

Figure 25 shows the microscopic morphology of the micro-grooves surface under different machining methods. It can be seen that there are more large-volume pits on the surface of micro-grooves without electrophoresis assistance.

## 5. Conclusions

This paper proposes template-based electrophoretically assisted micro-ultrasonic machining for the micro-channel machining of hard and brittle material substrates. The template-based electrophoretically assisted micro-ultrasonic machining micro-channel machine tool was developed, and experiments were carried out using the developed machine tool. This machine tool can realize template-based electrophoretically assisted micro-ultrasonic machining and has developed a constant force control system, which can realize the one-time forming and machining of micro-channels and is suitable for the rapid mass production of fixed-shaped micro-channels. The designed working liquid tank has a leveling device, which can ensure the parallelism between the template tool and the workpiece and ensure that the overall depth of the micro-channel is consistent. The main conclusions are as follows:

1. The template-based electrophoretically assisted micro-ultrasonic machining micro-channel machine tool can effectively implement the micro-channel machining of hard and brittle materials. The hardware part of the machine tool includes a vertical sliding table, a micro three-dimensional motion platform, an ultrasonic vibration system, a pressure sensor, a working fluid tank, and a template tool. The software part of the machine tool uses LabVIEW for modular development, which includes: an initialization module, fast positioning module, constant force tool setting module, constant force control machining module, and real-time position display module.
2. The machining experiments of different micro-channel shapes show that: the step-shaped micro-straight groove with the smallest contact area with the workpiece has the highest machining rate.
3. The machining experiments of different material substrates of micro-channels show that: the efficiency of micro-channels on single-crystal silicon is better than that on soda-lime glass.
4. Compared with micro-ultrasonic machining, template-based electrophoretically assisted micro-ultrasonic machining can effectively reduce the surface roughness of micro-grooves and has a positive effect on the material removal rate.

## Figures and Tables

**Table 1:** Mechanical and electrical parameters of duralumin material.

Material	Conductivity (IACS)/%	Resistivity (Ω·m)	Density (g/cm^3^)	Yield Strength (MPa)	Tensile Strength (MPa)
Duralumin	41	0.0415	2.82	455	510

**Table 2:** Comparison of the surface roughness and material removal rate of different machining methods.

Methods	Ra (μm)				MRR (10^6^ μm^3^/min)
	1	2	3	Average	
With electrophoresis assistance	1.803	2.137	1.936	1.959	0.859
Without electrophoresis assistance	2.279	2.606	2.749	2.545	0.828

## Data Availability

Not applicable.
